# Supplementary material for: Transgenic Microalgae Expressing Double-Stranded RNA as Potential Feed Supplements for Controlling White Spot Syndrome in Shrimp Aquaculture
Source: Microorganisms. 2023 Jul 26;11(8):1893. doi: 10.3390/microorganisms11081893 (PMC10459155; doi:10.3390/microorganisms11081893)
Supplement: Supplementary file 1 [file microorganisms-11-01893-s001.zip › microorganisms-2514336-supplementary.pdf]

Supplementary Data

Transgenic microalgae expressing double-stranded RNA as potential feed supplements for controlling white spot syndrome in shrimp aquaculture

Patai Charoonnart[1,2]*, Henry Nicolas Taunt[3], Luyao Yang[3], Conner Webb[4], Colin Robinson[4], Vanvimon Saksmerprome[1,2] & Saul Purton[3]

[1]Center of Excellence for Shrimp Molecular Biology and Biotechnology (Centex Shrimp), Faculty of Science, Mahidol University, Bangkok 10400, Thailand

[2]National Center for Genetic Engineering and Biotechnology (BIOTEC), National Science and Technology Development Agency, Pathum Thani 12120, Thailand

[3]Department of Structural and Molecular Biology, University College London, London WC1E 6BT, UK

[4]Centre for Molecular Processing, School of Biosciences, University of Kent, Canterbury CT2 7NJ, UK

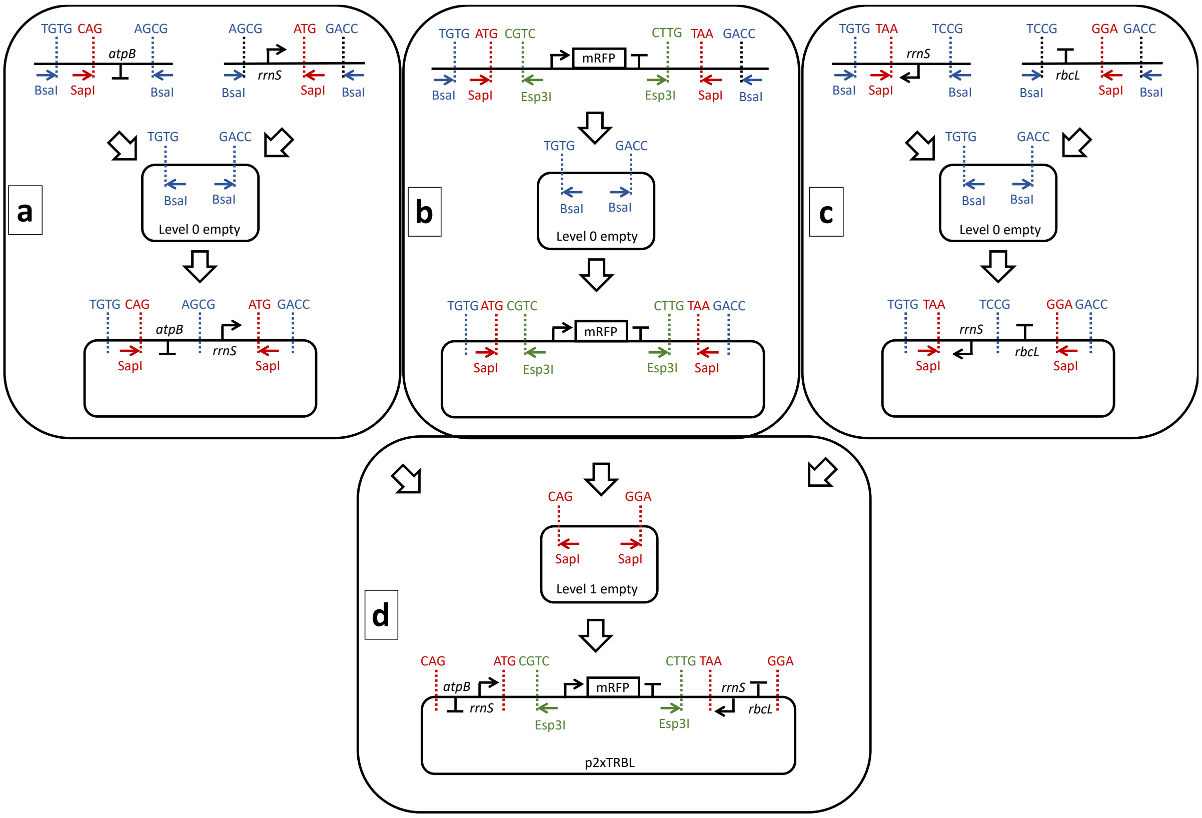

Figure S1 Golden Gate assembly of p2xTRBL. *C. reinhardtii* expression elements were amplified by PCR, with the addition of 5’ extensions to allow *Bsa*I cloning into the Level 0 empty vector (a, c). The mRFP cassette was synthesized *de novo*, also with the addition of Golden Gate cloning sequences (b). The three Level 0 cassettes were cloned into a Level 1 empty vector using *Sap*I to generate p2xTRBL (d).

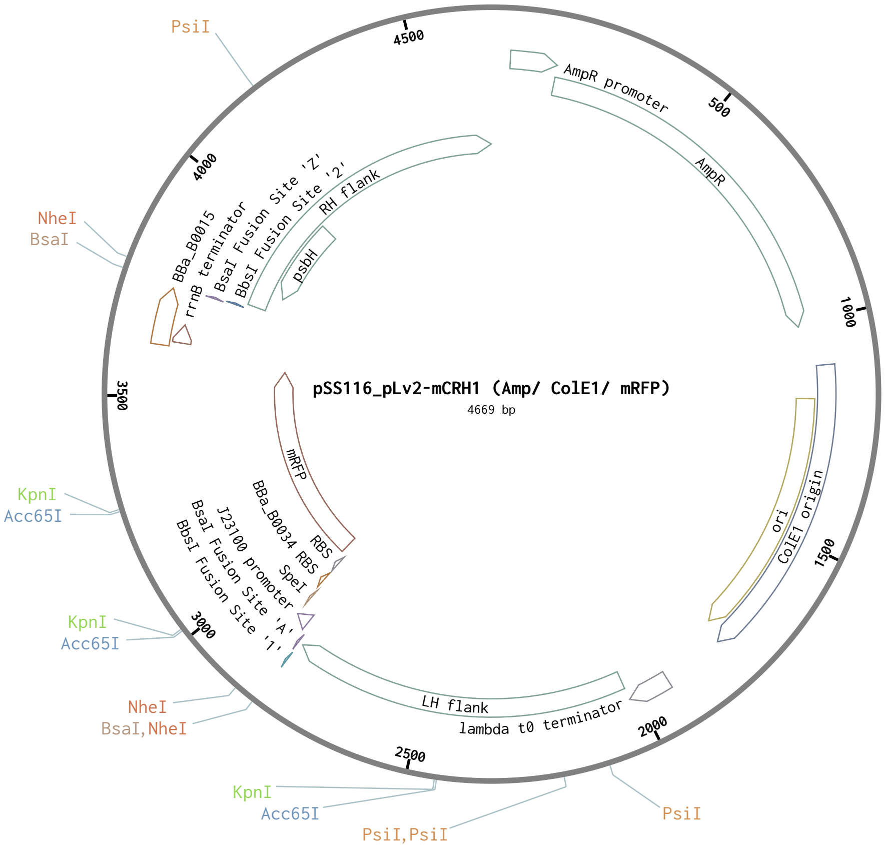

Figure S2 Map of plasmid pSS116, an integration vector for targeting transgenes into the *C. reinhardtii* chloroplast genome between *psbH* and *trnE2*.

Tataatgtatgtgtaattaaaataaattggctctttaagaagaaaacaacttaatggtgtcc
aaatatttattttaattacacataaaaaattaaacggagcagcttgttattgaaattttatt
aaaaaaaaaataaaaatttgacaaaaaaaaataaaaaagttaaattaaaaacactgggaatg
ttctacatcaatg**cgtc**gatctttctttcactctttcggtcgtgtcggccatcctcgccatc
actgctgtgattgctgtatttattgtgatttttaggtatcacaacactgtgaccaagaccat
cgaaacccacacagacaatatcgagacaaacatggatgaaacctccgcattcctgtgactg
ctgaggttggatcaggctacttcaagatgactgatgtgtcctttgacagcgacaccttgggc
aaaatcaagatccgcaatggaaagtctgatgcacagatgaaggaagaagatgcggatcttgt
catcactcctgtgg**cttg**taatgatgtagaacattcccagtgttttaatttaacttttta
tttttttttgtcaaattttatttttttttttaataaaatttcaataacaagctgctccgttt
ttatttttcatgatgtttatgtgaatagcataaacatcgtttttatttttatggtgtttagg
ttaaatacctaaacatcatt

Figure S3 Sequence of the *VP28* transcription cassette. The *VP28* sequence is highlighted in yellow. *Esp*31 fusion sites are in **bold**. The *rrnS* promoter elements are highlighted in green with the transcriptional start site indicated by a black triangle. The *atpB* terminator is highlighted in light grey and the *rbcL* terminator in dark grey. Inverted repeat regions are indicated by horizontal coloured arrows and the transcript termination sites by vertical black arrows.

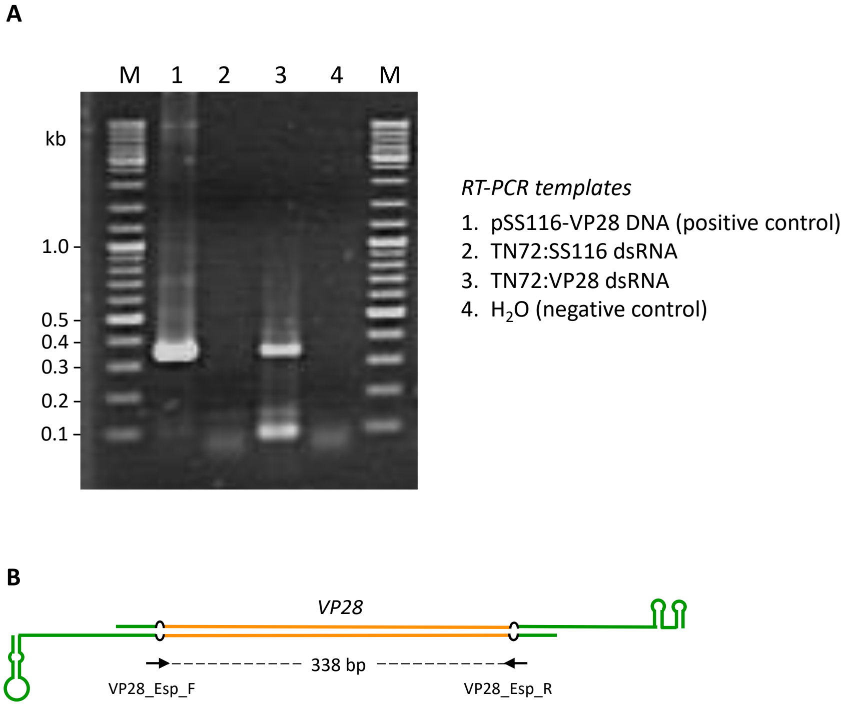

Figure S4 RT-PCR analysis of dsRNA from transformant TN72:VP28. **A**. Lane 1: PCR amplification of VP28 DNA from pSS116-VP28 plasmid. Lane 2: RT-PCR using dsRNA fraction from strain TN72:SS116. Lane 3: RT-PCR using dsRNA fraction from strain TN72:VP28. 4 RT-PCR using ddH_2O as template. **B**. Illustration of primers used for RT-PCR.

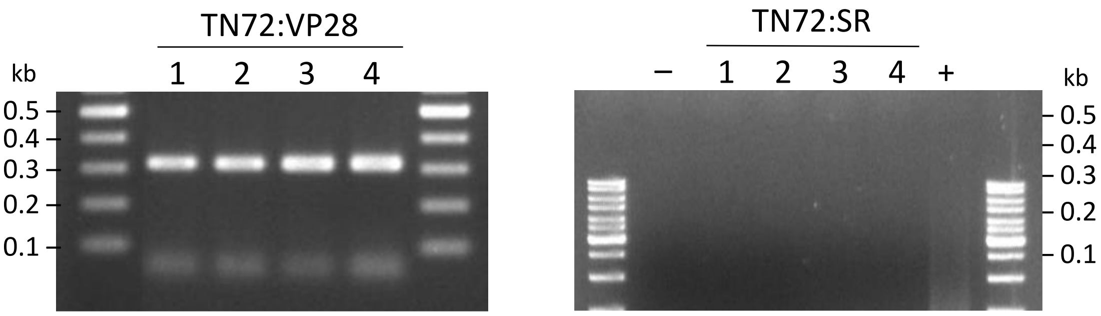

Figure S5 Detection of dsRNA from freeze-dried *C. reinhardtii*. Four replicate samples each representing 0.1 g of biomass from either TN72:VP28 (left panel) or TN72:SR (right panel) were used for dsRNA extraction and RT-PCR using specific primers VP28_Esp_F and VP28_Esp_R (See Figure S4). Controls are H_2O (–) or purified dsRNA (+) as template.

Table S1 Details of primers used

**Primer**	**Sequence (5'–3')**	**Function**	**Reference**
VP28_Esp_F	ATACGCGTCTCTCGTCGATCTTTCTTTCACTCTTTCG	Generation of *VP28* fragment for p2XTRBL cloning (*Esp*31 sites underlined). RT-PCR analysis of dsRNA from *E. coli* and *C. reinhardtii* (PCR product of 338 bp)	This work
VP28_Esp_R	ATACGCGTCTCTCAAGCCACAGGAGTGATGACAA		
rbcL_F	GTCACCACCAGACATACGAAG	Internal control for chloroplast DNA extraction. (PCR product of 264 bp)	[18]
rbcL_R	GGTCACTACTTAAACGCTAC		
TN72_F	GTCATTGCGAAAATACTGGTGC	TN72 plastome-specific primers for assessing homoplasmic status. (PCR product of 0.88 kb)	[19]
TN72_R	CGGATGTAACTCAATCGGTAG		
WSSV447_F	ATGAGAATGAACTCCAACTTTAA	WSSV load assay.	[26]
WSSV447_R	CAGAGCCTAGTCTATCAATCAT		
